# Online Respondent-Driven Sampling for Studying Contact Patterns Relevant for the Spread of Close-Contact Pathogens: A Pilot Study in Thailand

**DOI:** 10.1371/journal.pone.0085256

**Published:** 2014-01-08

**Authors:** Mart L. Stein, Jim E. van Steenbergen, Charnchudhi Chanyasanha, Mathuros Tipayamongkholgul, Vincent Buskens, Peter G. M. van der Heijden, Wasamon Sabaiwan, Linus Bengtsson, Xin Lu, Anna E. Thorson, Mirjam E. E. Kretzschmar

**Affiliations:** 1 Julius Center for Health Sciences and Primary Care, University Medical Center Utrecht, Utrecht, The Netherlands; 2 Centre for Infectious Disease Control, National Institute for Public Health and the Environment, Bilthoven, The Netherlands; 3 Centre for Infectious Diseases, Leiden University Medical Centre, Leiden, The Netherlands; 4 Department of Microbiology, Faculty of Public Health, Mahidol University, Bangkok, Thailand; 5 Department of Epidemiology, Faculty of Public Health, Mahidol University, Bangkok, Thailand; 6 Faculty of Social and Behavioural Sciences, University Utrecht, Utrecht, The Netherlands; 7 Southampton Statistical Sciences Research Institute, University of Southampton, Southampton, United Kingdom; 8 Faculty of Communication Arts, Chulalongkorn University, Bangkok, Thailand; 9 Department of Public Health Sciences, Karolinska Institutet, Stockholm, Sweden; 10 College of Information System and Management, National University of Defense Technology, Changsha, China; Kliniken der Stadt Köln GmbH, Germany

## Abstract

**Background:**

Information on social interactions is needed to understand the spread of airborne infections through a population. Previous studies mostly collected egocentric information of independent respondents with self-reported information about contacts. Respondent-driven sampling (RDS) is a sampling technique allowing respondents to recruit contacts from their social network. We explored the feasibility of webRDS for studying contact patterns relevant for the spread of respiratory pathogens.

**Materials and Methods:**

We developed a webRDS system for facilitating and tracking recruitment by Facebook and email. One-day diary surveys were conducted by applying webRDS among a convenience sample of Thai students. Students were asked to record numbers of contacts at different settings and self-reported influenza-like-illness symptoms, and to recruit four contacts whom they had met in the previous week. Contacts were asked to do the same to create a network tree of socially connected individuals. Correlations between linked individuals were analysed to investigate assortativity within networks.

**Results:**

We reached up to 6 waves of contacts of initial respondents, using only non-material incentives. Forty-four (23.0%) of the initially approached students recruited one or more contacts. In total 257 persons participated, of which 168 (65.4%) were recruited by others. Facebook was the most popular recruitment option (45.1%). Strong assortative mixing was seen by age, gender and education, indicating a tendency of respondents to connect to contacts with similar characteristics. Random mixing was seen by reported number of daily contacts.

**Conclusions:**

Despite methodological challenges (e.g. clustering among respondents and their contacts), applying RDS provides new insights in mixing patterns relevant for close-contact infections in real-world networks. Such information increases our knowledge of the transmission of respiratory infections within populations and can be used to improve existing modelling approaches. It is worthwhile to further develop and explore webRDS for the detection of clusters of respiratory symptoms in social networks.

## Introduction

For important respiratory pathogens, like influenza, SARS and tuberculosis, spatial proximity between social contacts is a major determinant in the transmission process [1]. To understand the dynamics of transmission of pathogens through a population, information on contact patterns is needed. First explorations to quantify contact patterns relevant for respiratory infections using contact diary questionnaires were conducted by Edmunds et al. in 1997 [2]. Subsequently, many other studies were performed in which the distribution of contacts was analysed among different age-groups [3], settings and across countries [4], [5]. Various study designs were applied and tested for performance and validity [6]–[9]. Up till now, most of these studies focused on recording and analysing contact patterns of randomly sampled individuals. Surveys were ‘egocentric’ and contained only self-reported information about characteristics of contacts of respondents and the links between them. Other studies investigated participants wearing digital devices that sense their proximity to others including their spatial movements [10], [11]. However, such studies can only be performed in specific settings and with small numbers of participants.

The rate at which infections spread across a community depends, among others, on the topology of the contact network [12]. Theoretically, it has been shown that network properties like the clustering of contacts and heterogeneity in ‘degree’ (i.e. the total number of contacts per individual) influence transmission dynamics [13]–[16]. For designing optimal control strategies, it is important to have knowledge on the network properties that allow or enhance the spread of infections along links in a network of individuals. More specifically, it might be advantageous to identify individuals who act as a bridge between communities or as hubs spreading to many other individuals.

Here we report on a pilot study using respondent-driven sampling (RDS) to recruit respondent as well as their close social contacts into a survey to study transmission related contact patterns. RDS is a variant of chain referral sampling, which includes ascertainment of degree distributions [17]. Different from snowball sampling, researchers keep track of who recruited whom and their numbers of social contacts. RDS is predominantly used for making prevalence estimations of characteristics in otherwise hard-to-reach populations such as injecting drugs users [18]–[20]. In this study we explored the technical feasibility and implementation of webRDS in a general population. We employed RDS with a different aim, namely as a sampling tool to study contact patterns relevant for the transmission of respiratory pathogens.

Increasing access to the internet, especially in low- and middle-income countries, offers new opportunities for epidemiological research [21]–[24]. Earlier studies found that social mixing patterns can be measured through simple internet-based surveys [6], [25]. Until now, only few studies used the internet for RDS (webRDS) [26]. A recent study performed in VietNam among internet-using MSM (‘men who have sex with men’) showed the potential of webRDS for sampling hidden and stigmatized populations [27]. The applicability of (web-)RDS for recording contacts relevant for respiratory-transmitted infections has, to our knowledge, not yet been investigated. We analysed the applicability of online social networks (i.e. Facebook) for inviting contacts, and the use of non-material incentives to stimulate recruitment. Secondly, we studied the correlations between individuals linked by recruitment chains and the distribution of connected components of the recruitment trees. Finally, we investigated whether webRDS can be used to detect clusters of influenza-like-illness symptoms in social networks.

## Materials and Methods

### Respondent Driven Sampling

RDS begins with the selection of initial respondents, called “seeds”. The seed is asked to complete a survey and afterwards provided with a limited number of coupons (usually three or four) to invite contacts who are then asked to do the same. Limiting the number of coupons for each participant forces the sample recruitment chain to penetrate into the social network of seeds. This process continues in recruitment ‘waves’, either until the desired sampled size is reached or until the distribution of participants' characteristics has stabilised between waves or until chains go extinct, ensuring that the final sample is not biased by the choice of the seeds [17].

### Web based RDS survey system

We developed a web based RDS survey system for facilitating and tracking online recruitment, based on a system that was implemented earlier by Bengtsson and colleagues [27]. We designed templates for invitation and reminder emails, which contained a link that provided direct access to the survey. These unique links were based on personal codes and automatically generated by the system for each participant. Each link could only be used once for filling in the survey to prevent repeated participation and participants seeing each others answers, and to control the number of friends that could be recruited. The questionnaire was divided over multiple pages (maximum of four questions per page). All text was provided in both Thai and English.

After submission of answers, respondents were redirected to a page for inviting contacts. We provided three options for inviting contacts: (1) sending an invitation email directly by the system to contact persons for whom email addresses were provided or (2) receive four separate invitation emails that could be forwarded to contact persons or (3) connect to Facebook to invite Facebook friends with a private Facebook message. For the first two email options the name of the recruiter was required, which appeared in the subject line of the email to personalise the invitation. As with the emails for seeds, the template of the invitation emails used for recruitment were standardised and contained background information about the project, a (new) unique link to the questionnaire, and a personal code. All emails and the first page of the questionnaire contained a link to unsubscribe for the survey, which led to another page on which reason(s) for not participating could be provided.

For inviting friends on Facebook, participants needed to login to their personal account, followed by accepting our app. The app automatically created Facebook private messages, each containing a unique link to the survey that could be forwarded one by one. As with option 2, there was a possibility for the recruiter to add personal text to each message, next to the invitation text provided by us.

### Illustration of network trees

As a non-material incentive, the progression of each network tree could be followed by the respondent on the accompanying institute website. Each network tree started with the code of the seed, which was linked to the corresponding codes of the recruited contacts. These contacts were each linked to contacts of contacts, and so on. The trees were anonymous and did not contain information provided by participants in the survey. All participants were referred to the institute website after sending out invitations. The network trees were updated daily and participants could return to the institute website at any moment.

### Informed consent and privacy

Apart from recruitment, the survey was anonymous. The questionnaire pages were preceded by an informed consent page containing the research purposes, information on data security, subjects' privacy, confidentiality (e.g. data was not shared with any third parties) and non-material incentive, and the contact details of the researcher. Participation was voluntary and individuals could withdraw at any moment during the survey by closing the browser. Only after accepting the form, individuals could proceed to the questionnaire.

More detailed information about the research project was provided in a separate link, which could be accessed at any time during the survey. Furthermore, the logos of the participating research institutes were displayed on every page of the survey (as well as in every invitation email), and were linked to the associated websites. The system converted IP addresses to a unique anonymous code using a one-way encryption algorithm; the original IP addresses were deleted. All communication between the user and the server was encrypted. The online database was also encrypted and password protected.

We obtained ethical approval from the Medical Ethical Committee from both the Faculty of Public Health Mahidol University (Thailand) and University Medical Centre Utrecht (The Netherlands).

### Study design, seeds, and setting

The developed webRDS system was applied for conducting contact diary questionnaires in Thailand between November 2012 and February 2013. As seeds we approached students from two Bangkok universities with an invitation email. Students, in physical group meetings varying between 6 and 30 persons, were first informed with a short oral presentation about the project, and afterwards contacted with an invitation email. In addition, respondents could become friends with the researcher on a Facebook page that was developed for this project, where updates on the network trees were presented and where participants could suggest friends as seeds (i.e. other than those personally approached).

The seeds were selected from a convenience sample of students, given sufficient spread of different curricula and academic years, in collaboration with lecturers and faculty deans of student affairs to prevent too much immediate overlap among contacts recruited by the seeds. Each seed was asked to recruit four close contacts (e.g. friends, family members and/or colleagues) whom they had met (according to the contact definition described below) in real-life in the past seven days. The time span of seven days was decided as it was a reasonable period for remembering close contacts and a seven days period includes the generation time of influenza [28], [29]. Each participant was provided with an invitation for each of their four contacts. In principle, there were no exclusion criteria; however, a contact had to have access to the internet. We sent out reminder emails to seeds and contacts who did not respond within two weeks after sending the invitation email.

Thailand has around 65.9 million inhabitants (20.5 million households, with an average size of 3.2 persons), of which 8.3 million are registered in the densely populated capital Bangkok. In 2012, 27.5% of the Thai population in rural areas (aged six years and older) was using a computer, 20.5% the internet and 66.2% a mobile phone. Bangkok has higher proportions of users: 44.4%, 51.5% and 84.0% respectively. The age groups of 6–14, 15–24 and 25–34 use the internet most frequently (46.5%, 54.8%, and 29.7% respectively within each age group). Internet-use is much lower for ages of 35 years and above [30]. In May 2013, Thailand counted around 18.5 million Facebook accounts of which most users were between the ages of 18–34 years [31].

### Online questionnaire

We asked participants to record the number of contacts they had during one full day (namely ‘yesterday’). A contact was defined as a person standing or sitting close – defined as within reach of an arm's length [7], [32] – to the participant for 30 seconds or longer. This space within arm's length was denoted as ‘YourSpace’. The definition was illustrated with pictures to clearly indicate which contacts should be recorded.

To limit the burden for each participant, the online questionnaire was kept short; in total it consisted of eleven questions. Participants were asked to record the number of contacts while travelling (e.g. train, metro, bus, shuttle boot, minibus, car, tuk-tuk) and at different locations (e.g. home, work, school/university, restaurant, coffee shop, sport/leisure, concert or other places). For the different locations, participants were asked to specify for each contact whether this person was younger than, the same age or older than the participant.

In Thailand, it is custom to share food with friends, family and/or colleagues. Therefore we asked for the number of contacts (within arm's length) with whom participants had breakfast, lunch, dinner and/or a snack break. In addition, we included a question on the number and age (specified in age groups) of contacts that lived in the household in the past seven days. To facilitate participation, participants were instructed to leave answer options empty when these were not applicable to them (instead of having to fill in a zero). Empty cells were treated as a zero during analyses for all participants who reached the last page of the questionnaire. The following basic demographic information was also collected for each participant: gender, age, educational level and postal code. We also asked participants to report any influenza-like-illness (ILI) symptoms (provided in a list) that they and/or their household contacts experienced in the past seven days.

### Analyses

Degree was defined as the sum of the numbers of contacts while travelling and at different locations reported by each respondent for one day. We censored degree and number of contacts while eating to a maximum of respectively 500 and 75 contacts per day for each respondent, which were considered as highest reasonable values. We fitted a negative binomial distribution to the observed degree distribution using maximum likelihood estimation (see Text S1).

The tendency of individuals in a network to be linked to similar individuals (‘assortative’ mixing, and vice versa ‘disassortative’ mixing if links are made between dissimilar individuals) can be measured by correlation coefficients between pairs of individuals [33]. To investigate mixing patterns within our sample, we calculated correlations between the recruiter and his/her recruited contacts. We used Pearson's *r* for integer variables (e.g. age, degree, household size, contacts while eating, and number of self-reported symptoms). We used phi coefficient (*r_φ_)* for binary variables (e.g. gender and two-or-more self-reported symptoms), and Spearman rank-order (*r_rank_*) for ordinal variables (e.g. educational level). For conducting null hypothesis tests for Pearson's *r*, a bivariate normal distribution is assumed [34]. Count variables were therefore log transformed, and bivariate normality was visually assessed using joint probability distribution plots (see Text S2).

Besides studying the correlation between characteristics of neighbouring nodes, RDS allows for comparison of individuals' characteristics across more than one link in the network. Investigating such correlations shows whether correlations seen for pairs of directly linked nodes persist beyond the first link. We calculated correlations for all possible link distances between respondents in the same component.

In RDS, respondents recruit contacts they know, with the result that respondents have similar characteristics. Thus, characteristics of respondents in the same component are correlated, and this affects the standard errors of survey estimates. A measure for this correlation is the intraclass correlation (ICC), derived in multilevel analysis [35]. The ICC can be interpreted as the expected correlation between two randomly chosen individuals within the same component. The ICC for a two-level model is defined as:
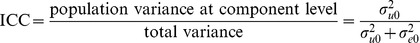
where the numerator is the variance at the component level 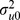, and the denominator represents the total variation in the model, which includes the variance at the component level and the variance at the respondent level 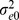. The ICC can be calculated for each variable separately and varies between 0–1. When the ICC is zero, observations can be considered independent (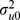 = 0). An ICC of 1 indicates that respondents in the same component respond identically (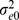 = 0). The higher the ICC, the less representative the sample is for the population given the variable considered and the sample size. Variance estimates were derived from an intercept-only multilevel model with restricted maximum likelihood estimation [35].

In the supplementary materials we analysed which variables are important in the recruitment process using logistic regression analyses (see Text S3). Furthermore, we explored whether correlations for age, gender and education between recruiters and their contact persons were only dependent on the direct recruiter (i.e. one step away, a first-order Markov assumption), by using a Monte Carlo technique to simulate a first-order autoregressive process (see Text S4). We also visually assessed equilibrium for all variables and applied the Volz-Heckathorn estimator [36] to estimate population proportions from our sample (see Text S5).

**Figure 1:**
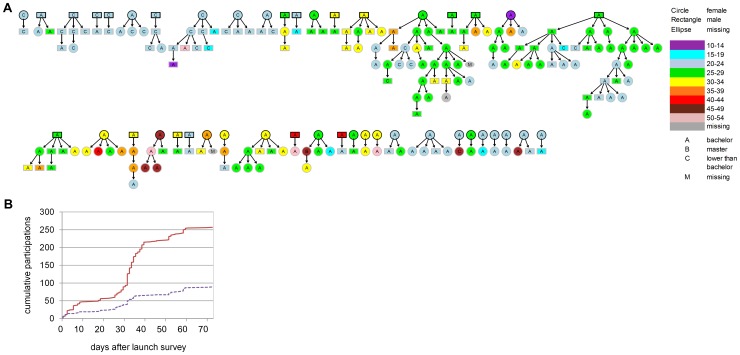
RDS recruitment. (**A**) RDS network trees showing age, gender and educational level of respondents. Only trees with two or more participants were displayed (in total 44 trees), symbols with black borders indicate seeds. (**B**) Cumulative number of participants and seeds (those who filled in the survey) over length of time the survey was active (in days), since the survey launch. The cumulative number of respondents is indicated by the red solid line, and participating seeds with the purple dotted line. Initially 44 students were approached, after 25 days more students were approached with an invitation email.

Analyses were performed with R (version 2.5.3); Figure 1 was created with the package Rgraphviz. RDS data file is available online, doi: 10.6084/m9.figshare.860458. We are currently improving the user interface of the webRDS system, researchers interested in the survey system are welcome to contact the authors.

## Results

### Study participants and recruitment waves

In total, we invited 191 students. Of those students, 89 (46.6%) entered the online survey using the personal link provided in the invitation email and 80 (41.9%) completed the online questionnaire, out of which 44 (23.0%) students invited contacts. Thirty-nine (20.4%) of these led to completion of at least one new questionnaire. The maximum number of recruitment waves was six. There were 15 (34.1%) network trees with two or more waves (Table S1). The two largest network trees consisted in total of 30 and 34 participants (Figure 1).

In total 257 individuals entered the online survey, of which 220 (85.6%) completed the survey. The age range of respondents was 14–52 years (mean age 26.7 years; standard deviation [SD] 6.73). There were 157 (61.6%) female and 98 (38.4%) male participants. Regarding education, 160 (63%) respondents had a bachelor degree, 53 (21.0%) had a master degree, and 41 (16.0%) had a junior high school, high school or higher diploma. When plotting the sample proportion by increasing sample size, we observed that the sample composition regarding age, gender and education did not stabilise (see Text S5). Most respondents (122, 47.5%) provided a postal code of a district outside Bangkok, 95 (37.0%) indicated to live in Bangkok, and 40 (15.5%) did not provide their postal code (see Table 1).

**Table 1:** Number of recorded contacts by different characteristics.

Category	Covariate	Number of participants	Mean (median;SD) number of reported contacts while travelling	Mean (median;SD) number of reported contacts at different locations
Age of participant	14–19	10 (3.9%)	61.4 (5.0; 99.8)	212.7 (144.0; 200.6)
	20–29	180 (70.0%)	17.3 (8.0; 33.0)	36.8 (17.0; 58.3)
	30–39	50 (19.5%)	16.4 (5.0; 8.3)	50.2 (15.5; 97.1)
	40+	13 (5.2%)	24.8 (2.0; 68.3)	42.3 (15.0; 69.0)
	Missing value	4 (1.6%)		
Gender participant	Female	157 (61.1%)	19.4 (7.0; 41.9)	42.0 (16.5; 75.8)
	Male	98 (38.1%)	18.3 (5.0; 38.5)	51.1 (18.0; 88.2)
	Missing value	2 (0.8%)		
Educational level participant	Master degree or higher	53 (20.6%)	16.7 (5.0; 42.3)	37.0 (15.5; 53.9)
	Bachelor degree	160 (62.3%)	20.7 (5.0; 44.8)	48.3 (15.5; 92.2)
	Lower than bachelor degree	41 (16.0%)	14.8 (11.0; 13.3)	45.2 (33.0; 63.1)
	Missing value	3 (1.2%)		
Household size	1	45 (17.5%)	12.6 (7.0; 22.3)	27.9 (14.0; 44.0)
	2	21 (8.2%)	13.9 (9.0; 16.6)	20.2 (14.0; 16.9)
	3	36 (14.0%)	13.6 (7.5; 23.4)	28.3 (14.5; 37.7)
	4	45 (17.5%)	13.9 (5.0; 47.7)	34.5 (14.0; 66.9)
	5	32 (12.5%)	20.4 (7.0; 33.5)	36.5 (21.0; 36.8)
	6	15 (5.8%)	11.9 (9.0; 17.2)	47.2 (27.0; 52.4)
	7+	27 (10.5%)	46.6 (9.0; 77.2)	95.9 (28.5; 115.8)
	Missing value	36 (14.0%)		
Participant living in Bangkok^a^	Yes	95 (37.0%)	19.4 (8.0; 40.1)	38.6 (16.0; 66.5)
	No	122 (47.5%)	18.1 (5.0; 40.8)	38.9 (17.5; 58.5)
	Missing value	40 (15.5%)		

^a^ Most seeds provided a postal code from a district far away from Bangkok, however we assume that most of these students stayed in a student dorm in Bangkok during the study week.

Two invited contacts indicated that they did not participate in our survey as they had participated earlier as a seed. Repeated recruitment among contacts occurred for an additional seven times (e.g. individual first invited by seed and later also invited by friend from another tree), based on the number of duplicates in email addresses used for recruitment. One individual indicated not to be interested in the subject of the survey. Two individuals entered the questionnaire but did not provide any information.

### Recruitment options used

Facebook was the most frequently used option for recruiting contacts. Facebook was used by 116 (45.1%) respondents compared to 29 (11.3%) who used one of the two email options. A total number of 580 coupons were handed out. Of those, 168 (29.0%) actually entered the survey and 140 (24.1%) completed the questionnaire. This means that we obtained 140 pairs of linked individuals who both completed the survey. Of the successfully recruited contacts, 117 (83.6%) were invited by 63 respondents who used Facebook, compared to 23 (16.4%) contacts who received an email from their recruiter (Table 2). Seventy-five (29.1%) respondents did not use a recruitment option after finishing the questionnaire. See Table S1 for a detailed overview of the used recruitment options.

**Table 2:** Number of successful recruitments by recruitment option.

Recruitment option used	0 (‘no’)	1	2	3	4	Total successful recruitments (n = 140)
Facebook	53	35	10	10	8	117 (83.6%)
Indirectly email	8	5	1	0	0	7 (5.0%)
Direct email	7	3	3	1	1	16 (11.4%)

Successful recruitment (of 1 to max. 4 contacts) counts when the invited contact also completed the survey; 0 (‘no’) indicates that recruiter invited his/her contacts but these contacts did not complete the survey. 75 respondents did not invite anyone after filling the survey.

### Technical issues

Although the invitation emails were evaluated for spam content, a number of email providers blocked our emails. This severely affected participation rates in the first phase of the pilot. Furthermore, as personal codes could only be used once for completing the survey, participants could not return to the survey after closing the browser. Therefore, respondents who postponed recruitment were unable to recruit contacts at a later stage.

Facebook made it easier for respondents to recruit contacts, as they did not have to provide their email addresses. However, the Facebook ‘Send Dialog’ application (for sending private messages to friends) that was used is not supported for mobile devices. Therefore, the Facebook invitation option was either invisible when using a mobile device, or in some operating systems, the option was provided but gave an error when selected. With the Send Dialog the length of the standard invitation text that can be provided by the researcher is restricted, and in some occasions only partly displayed depending on the settings of the recipient. In addition, we were unable to send out reminders to contacts that had been invited via the online social network. The latter was possible for the two email recruitment options, but errors were sometimes made with filling in email addresses.

### Number of reported contacts

**Figure 2:**
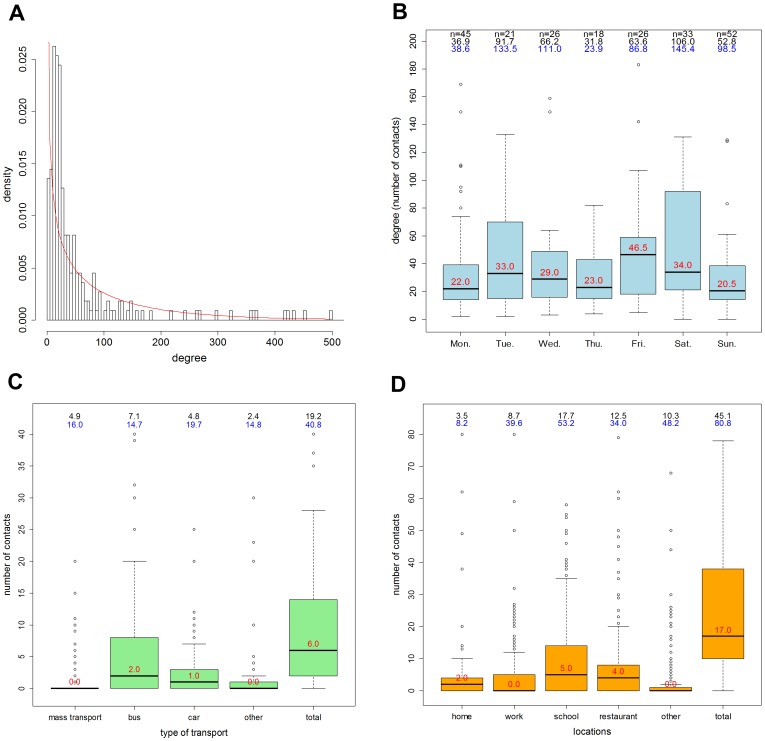
Recorded contacts. (**A**) Distribution of reported degree, the line indicates the fitted negative binomial distribution; (**B**) degree by day of the week (outliers >200 are not shown); (**C**) contacts while travelling with mass transport (sky train, subway and/or airplane), bus/minibus/shuttle boot, car/taxi and/or motorbike/tuk-tuk (outliers >40 are not shown); (**D**) numbers of contacts at different locations (outliers >80 are not shown). School was defined as ‘school/university’, ‘restaurant’ includes contacts at coffee shop, and ‘other’ is the sum of contacts encountered at sport/leisure, concert and ‘other places’. Above each plot in B, C and D the mean and SD (in blue) are displayed and within each plot the median (in red); B also contains the number of observations. Plots in C and D are based on an equal number of observations (n = 221).

A total of 19501 contacts were recorded, ranging from 0 to 4456 contacts per respondent per day. Three respondents reported more than 500 contacts. Figure 2 displays the degree distribution and the best fitting negative binomial distribution (mean = 88.2; dispersion *k* = 0.57). Less contacts per respondent were reported while travelling (the sum of contacts while travelling with different transport vehicles) than for locations (the sum of contacts for all locations together), respectively 19.2 (median = 6, SD = 40.8) and 45.1 (median = 17, SD = 80.8) mean contacts per day. Of all recorded contacts, 7574 (55.0%) were made during weekdays, compared to 6205 (45.0%) contacts that were made during the weekend (degree was censored to a maximum of 500 contacts per respondent). On average a degree of 62.3 (median = 25, SD = 100.7) was reported per participant per day, with Friday and Saturday having the highest degree per person (median 46.5 and 34.0 respectively) and Sunday the lowest (median of 20.5).

### Contacts while travelling and at different locations

While travelling (Figure 2), on average most contacts were made in the bus, mini bus or shuttle boat (mean of 7.1 contacts per person). Remarkably, the mean number of contacts per person for car or taxi was not much lower (4.8). For mass transport (e.g. sky train, subway or airplane) the number of reported contacts per person was over-dispersed. Comparable patterns were seen for motorbike/tuk-tuk, which is probably due to the larger versions of tuk-tuks (which can carry >3 persons). Twenty-six (11.8%) respondents indicated that they did not use any transport vehicles on the specific recording day.

Figure 2d shows the average number of contacts reported per participant for different locations. Participants in the age class 14–19 reported the highest numbers of contacts for each location, except for work. Most contacts in this age class were reported for school/university and ‘other’ (e.g. sports/leisure, concert and other places) locations. For the oldest age class (40+), most contacts were made at work. Table 1 shows the number of contacts while travelling and at different locations for different respondents' characteristics. Regarding contact numbers while eating, most contacts were reported during lunch and dinner. Again, the age class 14–19 reported overall the highest averages (which is an average of the sum of contacts during breakfast, lunch, dinner and/or snack break), and the age classes 20–29 and 30–39 had more varied numbers of contacts (see Table S2).

### Link recruiter and recruited

**Figure 3:**
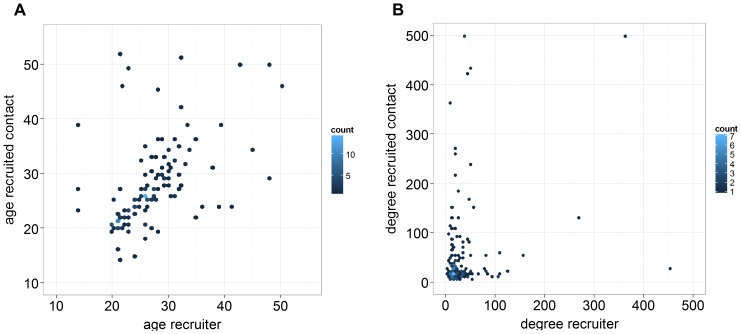
Correlations between recruiter and recruited contact. Graphs display correlations for (**A**) age, (**B**) degree (untransformed). Overlapping points were made visible with a colour scale.

We examined correlations for characteristics and numbers of contacts between linked respondents (recruiter versus recruited contact) (Table 3). Strong correlations were found for age (*r* = 0.555, *p*<0.001), gender (*r_φ_* = 0.205, *p* = 0.008) and education (*r_rank_* = 0.520, *p*<0.001). These positive correlations indicate that recruitment is assortative by age (Figure 3a), gender and educational level.

**Table 3:** Correlations between directly linked individuals in one component.

	Cor.	df	p value
Age (*r*)	0.555 [0.439 – 0.652]	163	<0.001
Gender (*r_φ_*)	0.205 [0.054 – 0.346]	164	0.008
Education (*r_rank_*)	0.520 [0.383 – 0.653]	164	<0.001
Degree log (*r*)	0.010 [−0.156 – 0.176]	138	0.907
Household size log (*r*)	0.058 [−0.110 – 0.222]	137^a^	0.499
Food with log (*r*)	0.215 [0.051 – 0.368]	138	0.011
Number of reported symptoms^b^ log (*r*)	−0.095 [−0.257 – 0.072]	138	0.266
Two or more reported symptoms^b^ (*φ*)	−0.060 [−0.223 – 0.108]	138	0.488

^a^ One case who reported >500 household contacts was removed.

^b^ Based on total number of self-reported symptoms by each respondent.

No assortativeness was observed by degree between linked nodes. Figure 3b shows the degree of the recruiter versus the degree of the recruited contact. The distribution corresponds with a random mixing in a population with negative binomially distributed degree (Figure 2a). The summary statistic between pairs of linked nodes indicated random mixing by degree (*r* = 0.010, *p* = 0.907). Comparable correlations were seen for household sizes of pairs of respondents (*r* = 0.058, *p* = 0.499). For numbers of contacts while eating a weak assortative tendency was seen (*r* = 0.215, *p* = 0.011). See Text S2 in supplementary materials for scatterplots.

### Successive links between contact persons in the same component

**Figure 4:**
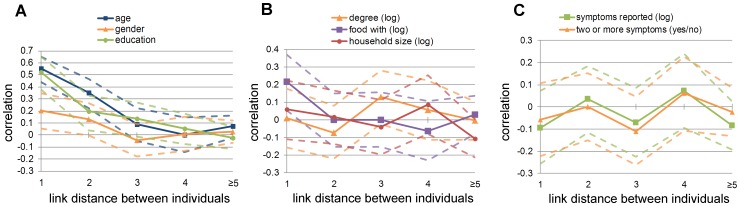
Correlations between any two individuals with different link distance. With graph **A** showing the correlations for age, gender, and education. Graph **B** displays the correlations in degree, number of contacts while having food, and household size (all after log transformation). Graph **C** shows the correlations for total number of self-reported symptoms (after log transformation), and for two or more self-reported symptoms (yes/no). Distances of five or more links were lumped together. The dotted lines show the confidence intervals.

Figure 4 shows the correlations between individuals across several steps in the recruitment chain. For age, gender and education the positive correlations decrease after the first link and disappear after a distance of three or more steps between any two individuals in the same chain (Figure 4a). For degree, numbers of contacts while having food, and household size, no correlations were observed over all distances (Figure 4b). Comparable analyses can be performed based on recruitment waves (i.e. only forward steps, see Text S4).

### Intraclass correlations

The ICC values for age, gender and education (respectively 0.470, 0.203 and 0.435) suggest a relatively large homogeneity for these characteristics within network components (Table 4). By contrast, low ICC values were found for log degree (0.064), numbers of contact while eating (0.142), household size (0), numbers of self-reported symptoms (0), and two or more reported symptoms (0). This confirms the results obtained through the correlations reported above in which we did not explicitly take interdependence of observations in components into account.

**Table 4:** Intraclass correlation coefficients.

	ICC	Variance between components ( 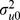 )	Variance within components ( 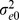 )
Age	0.470	22.848	25.812
Gender	0.203	0.049	0.203
Education	0.435	0.391	0.509
Degree (log)	0.064	0.087	1.281
Food with (log)	0.142	0.150	0.910
Household size (log)	0	0.000	0.511
Number of reported symptoms (log)	0	0.000	0.659
Two or more reported symptoms (yes/no)	0	0.000	0.210

### Self-reported symptoms

The mean number of self-reported symptoms was 1.8 (median = 2, SD = 1.5; varying from 0 to 8 symptoms per person). Of the respondents who completed the survey, 66 (30.0%) reported two-or-more symptoms, of which 7 (3.2%) persons reported a set that indicates flu-like symptoms (e.g. a combination of the symptoms fever, headache and muscle pain), and 13 (5.9%) reported common cold-like symptoms (e.g. a combination of the symptoms runny nose, sore throat and cough). Seventy-four (33.6%) respondents reported one-or-more household contacts with symptoms in the same period (Table S3). Random mixing was seen by total number of self-reported symptoms and by the variable two-or-more self-reported symptoms at all distances (Table 3 and Figure 4c).

## Discussion

We presented the results of a pilot study demonstrating the applicability of webRDS for studying contact patterns, especially those relevant to the transmission of airborne infections, of socially connected individuals. To our knowledge, this is the first online study in which RDS was applied to collect data specifically on human contact patterns relevant for close-contact pathogens, and the first in Thailand in which contact data was collected via the internet. By studying the correlations in characteristics between recruiter and recruited contacts, we found assortative mixing by age, gender and education, and random mixing by numbers of contacts. Building on earlier work in Vietnam [27], we have shown that webRDS can be used to elicit information about social contact networks and to collect empirical data on mixing patterns that are relevant for communicable disease transmission.

In contrast to most previous contact studies that focused on egocentric data [5], [8]–[11], [37], we used RDS to include contacts and contacts of contacts of the initial respondents into the study. This sampling method allows researchers to collect contact data in connected components of respondents, i.e. within the social structures where transmission actually occurs. In particular, mixing patterns and heterogeneity in numbers of contacts in social networks can be studied directly thereby providing information on possible transmission routes of communicable diseases. Assortative mixing by, for instance, age affects the spread of infections through a community. When individuals of a similar age class primarily have contact with each other, infections are likely to spread more within those subgroups. On a similar note, data on the distribution of degree within a community provides information on individuals who have many contacts and are therefore more likely to become infected and to infect others than individuals with fewer contacts. Theoretically it has been shown that highly over-dispersed degree distributions strongly affects the basic reproduction number, which is an important indicator of how fast an infection spreads and what fraction of the population will be infected [38]. Such RDS collected information on the contact network can be used as input for mathematical models to better describe transmission dynamics and impact of public health interventions, such as vaccination or isolation of certain groups within the population.

In contrast to previous webRDS studies [26], [27], [39], we have demonstrated that sampling can be performed without material incentives. Our non-material incentive was considered a fun motivator for recruiting contacts. Although the use of a monetary reward or a combination of incentives as was applied by Bengtsson et al. 2012 [27], could have increased the number of waves in our study, applying material incentives generally increases the risk of attracting ‘cheaters’ (e.g. respondents that participate multiple times by recruiting themselves to receive multiple rewards) that can severely affect study validity. In general, surveys in Thailand are performed without incentives as contributing to research is culturally considered an activity that does not require a monetary reward.

Our results underpin the importance of recruiting motivated and well-informed seeds, as was also seen in previous RDS studies. The use of a Facebook option for recruitment of friends has shown to be of critical importance for recruitment of contacts in the Thai study population. By providing recruiters with four private Facebook messages or invitation emails, we facilitated recruitment and gained more control over the sampling process, despite some technical challenges such as that the Facebook Send Dialog is not supported on mobile devices. Recruitment was more personal and directed, compared to the sharing of survey links in public areas as was done by Bauermeister et al. 2012 [39].

However, our results are based on a rather small sample and mainly represent students and their contacts of similar age (e.g. the age class of 20–35, see also Text S5). Several factors might have restricted recruitment and consequently the penetration of our survey into different layers of the Thai population, for example limited internet access, unfamiliarity with online recruitment of friends, and perceiving an invitation mail as spam. In addition, the criterion of recruiting only contacts that were seen in the past seven days may have contributed to limit sample size and composition. In case students and their contacts did not travel back to their parental home during the weekend, they were not likely to meet many other contacts outside the university and/or work.

In contact diary studies, differences in contact definition cause heterogeneities in numbers of recorded contacts and influence the assessment of the importance of settings in disease transmission risk [32]. Although the applied contact definition in our study was fairly simple and not limited to physical contact, for events like travelling by metro or bus during rush hours it is difficult to estimate the number of persons within arm's length. For these events reported numbers of contacts may be less reliable. For contacts that occur repeatedly within stable relationships such as within households, schools and workplaces, repeated measures on different days and asking recruited contacts about the contact they had with their recruiters (and vice versa) will provide insight in reporting bias and the validity of the data [8]. Moreover, repeated measures would aid participants in recalling daily contacts.

RDS is by nature subjected to clustering due to its sampling process. Clustering refers to how many of an individual's contacts, and subsequently how many of the contacts reported by individuals in the same referral chain, also had contact among each other. Clustering can have a profound effect on disease spread [16], [40]–[42]. For example, high clustering of contacts means more local spread and thus a rapid local depletion of susceptible individuals. In future surveys more information should be collected on repetitive recruitment among participants. Such information can be used to make estimates of clustering. Repeated recruitment could possibly be measured by asking respondents to report their personal code, with which they were initially invited to the survey, the moment they receive a second invitation (or more).

While RDS population estimates were not the aim of this study, we were interested in obtaining samples of respondent-contact pairs. Typically with RDS, well-connected individuals (i.e. high degree individuals) tend to be over-sampled because many recruitment paths lead to them. The bias that is introduced, with respect to number of contacts, is corrected for by the RDS estimators when making inferences to the population [43]. Increasing the number of coupons could provide a better view on an individual's entire contact structure, although it would increase the participation burden (e.g. more contacts have to be approached) and increase the probability of multiple recruitment.

In addition, with RDS respondents tend to recruit contacts who they think will participate, making the peer recruitment anything but random. For studying the representativeness of network links, more information is required about the (non-randomly) chosen contacts. For example, during future research recruited contacts can be asked to specify the relation he/she has with the recruiter, and to indicate the type, frequency and duration of contact, to learn more about the contact persons that are included in the sample. In addition, by defining more specific recruitment criteria link sampling could become more controlled in order to counter bias.

In future research, recruitment of seeds could be organised through online communities (e.g. panels [44] or online social networks), to capture a variety of seeds from all levels of the population. In general, participants from online panels are used to fill in web surveys, which will help researchers in the search for generative seeds. In addition, selecting seeds through the web might result in the inclusion of seeds from different geographical locations, which decreases the clustering among contacts. However, this requires that seeds can be motivated for peer-recruitment without researchers having to physically meet with these individuals. If possible, it will then also be interesting to explore the feasibility of using a probability based sample of seeds, i.e. selecting seeds randomly, thereby providing every individual in the population a chance of being selected as a seed. Representatively selected seeds for webRDS would retain the benefits of a random sample, such as collected in earlier egocentric studies, but are also likely to reach a more representative sample of the contact networks in a population. In theory, longer recruitment chains ensure that the sociometric distance between the seeds and the bulk of the sample will be large, hereby enhancing the diversity and representativeness of the sample [45]. Furthermore, the use of mobile devices for recruitment of contacts should also be further explored. Communication through smartphones or other mobile devices will continue to grow in Thailand and elsewhere providing new opportunities for webRDS research.

In principle, with webRDS it is possible to recruit a sample relatively fast compared to offline RDS [46] or traditional sampling techniques. In our pilot study, recruitment of additional seeds after day 25 led to a three times higher number of recruited participants (from around 50 participants to a sample size of over 200, see Figure 1b) within only 15 days. Although the pilot sample size was too small to include a large number of (linked) individuals with influenza-like-illness symptoms, the sampling speed of webRDS and the assortative recruitment by age, gender and education is potentially useful for reaching contacts at risk for infection, and for detecting clusters and studying the spread of respiratory agents in social networks at the level at which it is actually occurring. For example, webRDS could be applied for case-contact tracing where reported cases act as seeds, who are then asked to recruit contacts that they have physically seen during their infectious period. The benefit is that respondents are in control of recruitment, instead of health authorities, which is efficient as individuals know with whom they had contact and they can approach their contacts directly. In addition, with the use of the internet the tracing of contacts will be accelerated (compared to traditional methods) that will save time, human resources, and possibly provides the health authorities with options to intervene earlier during an outbreak (e.g. applying of interventions to control the spread) thereby preventing new cases.

Despite the methodological challenges, RDS allowed us to study connected components of individuals and obtain information about links within the network. Such information increases our understanding of contact networks relevant for the transmission of respiratory infections and can be used to improve existing modelling approaches. The application of webRDS for the purpose of studying contact patterns within real-life network structures is promising and will be explored further in future studies.

## Supporting Information

Figure S1 **Sample composition over waves for gender, education and age.** The plots above (**A**, **C** and **E**) display the cumulative proportions or averages over waves. The plots below (**B**, **D** and **F**) display the proportions or averages in each wave.
(PDF)

Figure S2 **Sample proportions and VH estimates with increasing sample size (not adjusted for network size) for all variables.** The solid lines indicate the raw sample proportions or average, and the dotted lines indicate the VH estimates. (**A**) educational level; (**B**) number of household members (categorised); (**C**) recruitment option used; (**D**) number of household members with symptoms (categorised); (**E**) age (integer); (**F**) male; (**G**) average number of contacts while eating (integer); (**H**) flu (combination of the self-reported symptoms fever, headache and muscle pain) and cold symptoms (combination of the symptoms runny nose, sore throat and cough); (**I**) average number of self-reported symptoms (integer); (**J**) average number of household members (integer).
(PDF)

Table S1 **Number of participants and used recruitment options over waves.**
(PDF)

Table S2 **The number of contacts while having food (mean, median, SD) younger, same age or older than participant.**
(PDF)

Table S3 **Reported symptoms.**
(PDF)

Text S1 **Fitting of a negative binomial distribution to degree.**
(PDF)

Text S2 **Links between recruiter and recruited contact person: descriptive statistics.**
(PDF)

Text S3 **Drivers of the recruitment process.**
(PDF)

Text S4 **Exploring the first–order Markov assumption.**
(PDF)

Text S5 **Sample composition, equilibrium curves and RDS estimates.**
(PDF)
