# Supplementary figures and images for: Online Respondent-Driven Sampling for Studying Contact Patterns Relevant for the Spread of Close-Contact Pathogens: A Pilot Study in Thailand
Source: PLoS One. 2014 Jan 8;9(1):e85256. doi: 10.1371/journal.pone.0085256 (PMC3885693; doi:10.1371/journal.pone.0085256)

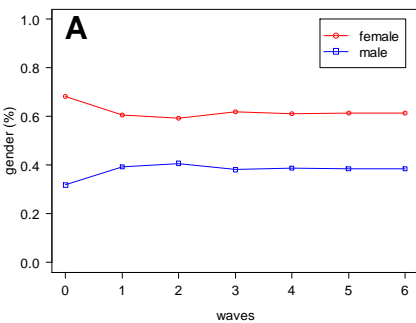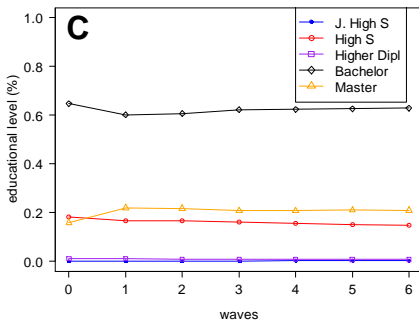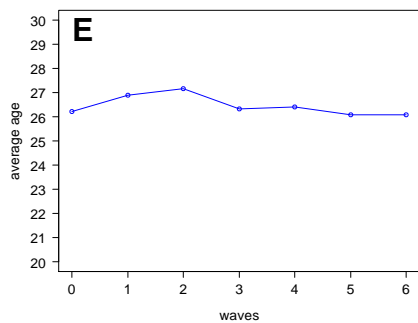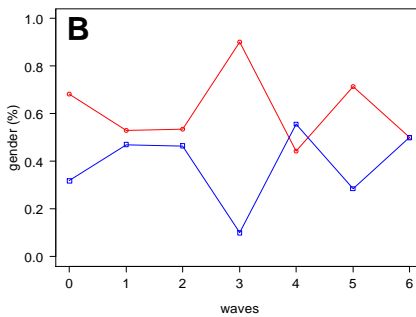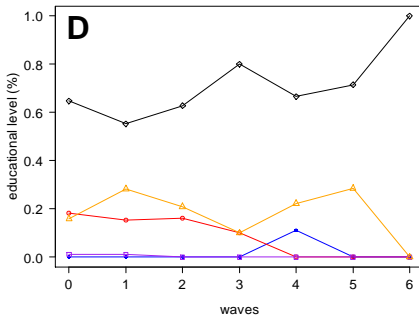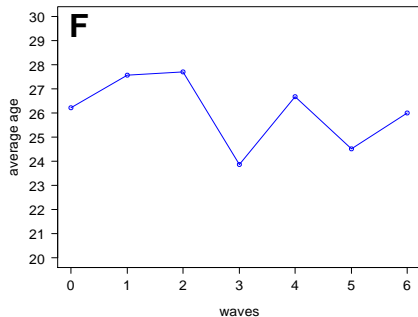

Supplement: Figure S1 — Sample composition over waves for gender, education and age. The plots above (A, C and E) display the cumulative proportions or averages over waves. The plots below (B, D and F) display the proportions or averages in each wave. (PDF) [file pone.0085256.s001.pdf]

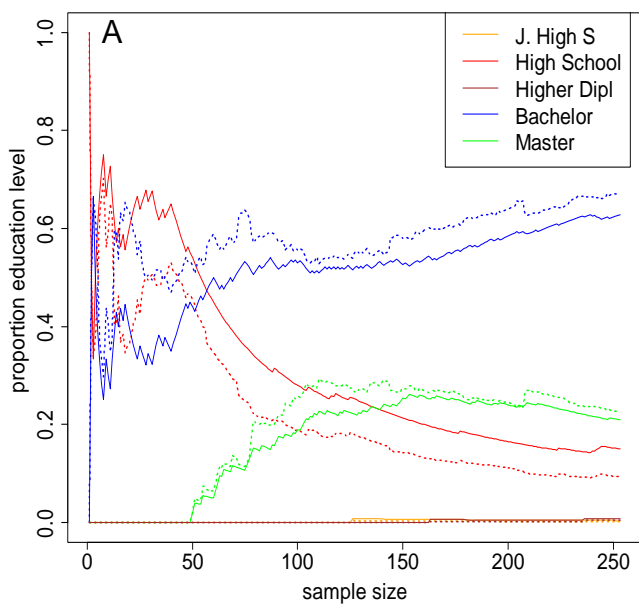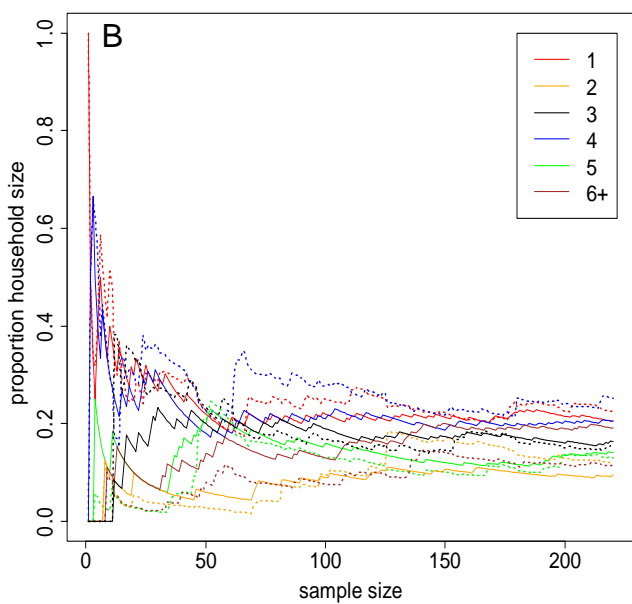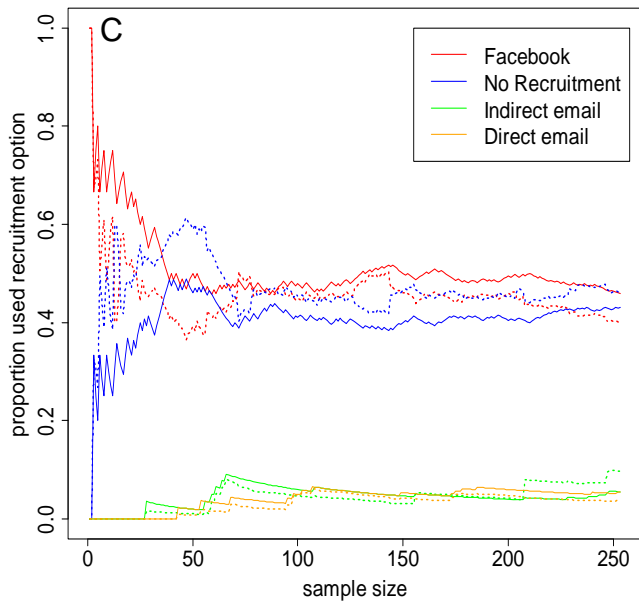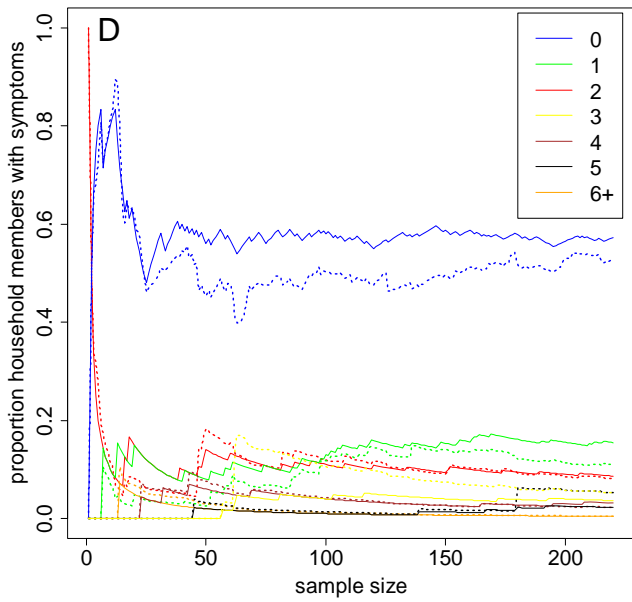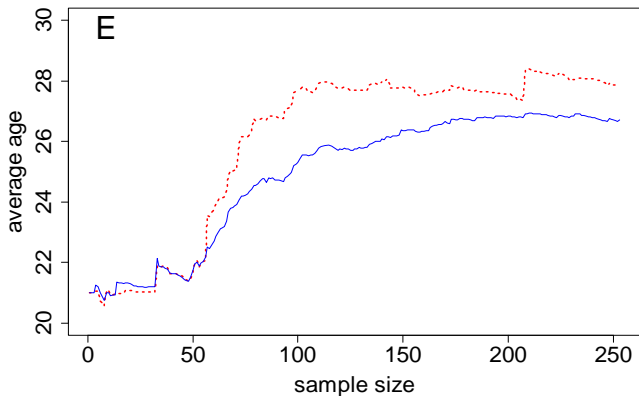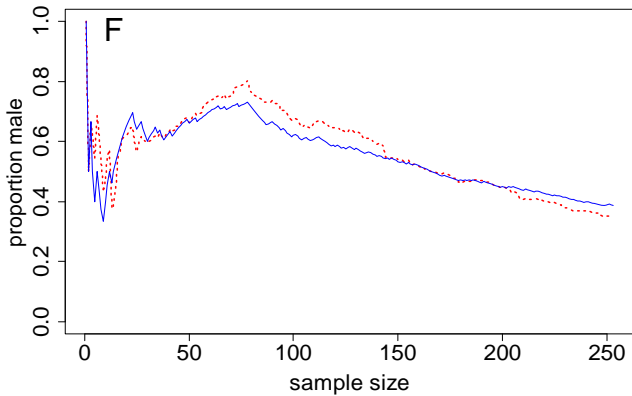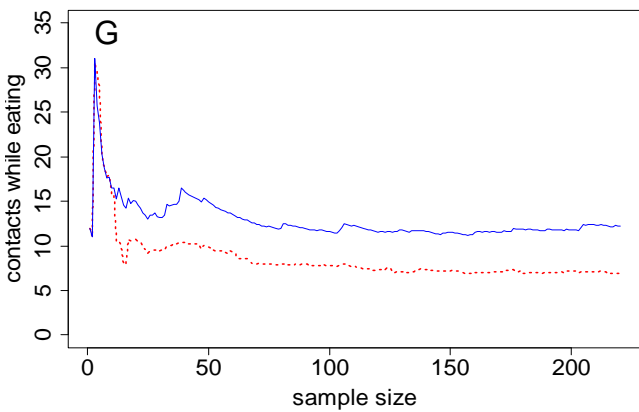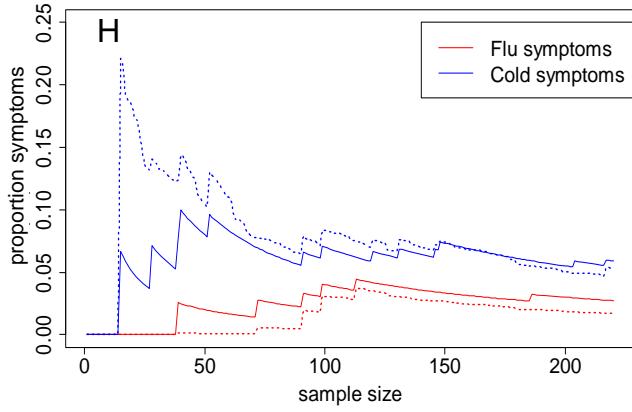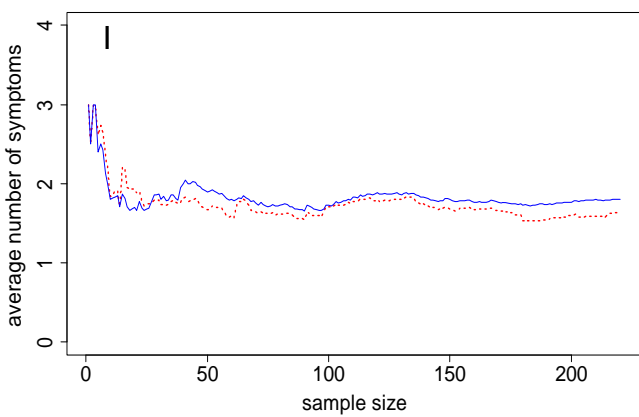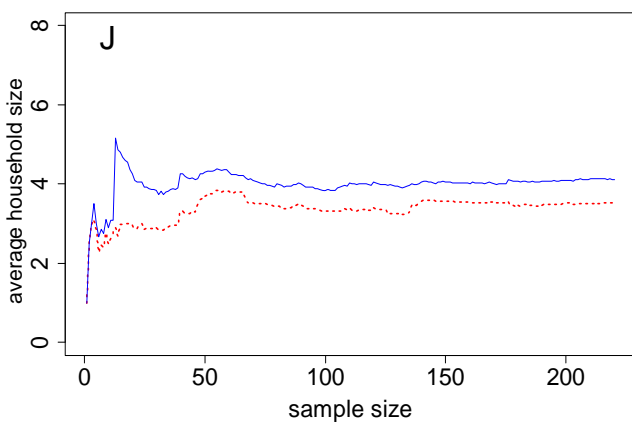

Supplement: Figure S2 — Sample proportions and VH estimates with increasing sample size (not adjusted for network size) for all variables. The solid lines indicate the raw sample proportions or average, and the dotted lines indicate the VH estimates. (A) educational level; (B) number of household members (categorised); (C) recruitment option used; (D) number of household members with symptoms (categorised); (E) age (integer); (F) male; (G) average number of contacts while eating (integer); (H) flu (combination of the self-reported symptoms fever, headache and muscle pain) and cold symptoms (combination of the symptoms runny nose, sore throat and cough); (I) average number of self-reported symptoms (integer); (J) average number of household members (integer). (PDF) [file pone.0085256.s002.pdf]
