# Supplementary material for: Online Respondent-Driven Sampling for Studying Contact Patterns Relevant for the Spread of Close-Contact Pathogens: A Pilot Study in Thailand
Source: PLoS One. 2014 Jan 8;9(1):e85256. doi: 10.1371/journal.pone.0085256 (PMC3885693; doi:10.1371/journal.pone.0085256)
Supplement: Table S1 — Number of participants and used recruitment options over waves. (PDF) [file pone.0085256.s003.pdf]

Table S1. Number of participants and used recruitment options over waves.

	Wave 0	Wave 1	Wave 2	Wave 3	Wave 4	Wave 5	Wave 6	Total
Number of trees with	45	29	10	3	0	0	2	89
Number of participants in	89	86	43	21	9	7	2	257
Number of incomplete responses (persons who provided DI[a])	9 (7)	12 (11)	6 (6)	7 (6)	2 (2)	1 (0)	0 (0)	37 (32)
Filled in during weekday[b] (% per wave)	65 (73.0)	61 (71.8)	35 (81.4)	17 (85.0)	7 (87.5)	2 (33.3)	1 (50.0)	188 (74.3)
Filled in during weekend[b] (% per wave)	24 (27.0)	24 (28.2)	8 (18.6)	3 (15.0)	1 (12.5)	4 (66.7)	1 (50.0)	65 (25.7)
Yesterday was a weekday[b] (% per wave)	55 (61.8)	57 (67.1)	29 (67.4)	9 (45.0)	5 (62.5)	3 (50.0)	1 (50.0)	159 (62.8)
Yesterday was a weekend day[b] (% per wave)	34 (38.2)	28 (32.9)	14 (32.6)	11 (55.0)	3 (37.5)	3 (50.0)	1 (50.0)	94 (37.2)
Recruited by email[c] (% per wave)	73 (82.0)	22 (25.6)	9 (20.9)	0 (0)	0 (0)	0 (0)	0 (0)	104 (40.5)
Recruited by Facebook[c] (% per wave)	0 (0)	64 (74.4)	34 (79.1)	21 (100)	9 (100)	7 (100)	2 (100)	136 (52.9)
Through registration page[c] (% per wave)	16 (18.0)	0 (0)	0 (0)	0 (0)	0 (0)	0 (0)	0 (0)	16 (6.2)
RO Facebook[d] (% per wave)	46 (51.7)	31 (36.0)	18 (41.9)	9 (42.9)	7 (77.8)	5 (71.4)	0 (0)	116 (45.1)
RO indirect email[d] (% per wave)	10 (11.2)	4 (4.6)	0 (0)	0 (0)	0 (0)	0 (0)	0 (0)	14 (5.4)
RO direct email[d] (% per wave)	9 (10.1)	4 (4.6)	2 (4.6)	0 (0)	0 (0)	0 (0)	0 (0)	15 (5.8)
Did not recruit (% per wave)	24 (27.0)	47 (54.7)	23 (53.5)	12 (57.1)	2 (22.2)	2 (28.6)	2 (100)	112 (43.6)

a) Participants provided demographic info (DI): gender, age and educational level, but no information on their contacts. **b)** Weekday is Monday to Friday; weekend is Saturday and Sunday. **c)** Includes respondents who did not complete the questionnaire. **d)** Recruitment option (RO) used by participant to recruit contacts.
