# Supplementary material for: Online Respondent-Driven Sampling for Studying Contact Patterns Relevant for the Spread of Close-Contact Pathogens: A Pilot Study in Thailand
Source: PLoS One. 2014 Jan 8;9(1):e85256. doi: 10.1371/journal.pone.0085256 (PMC3885693; doi:10.1371/journal.pone.0085256)
Supplement: Table S2 — The number of contacts while having food (mean, median, SD) younger, same age or older than participant. (PDF) [file pone.0085256.s004.pdf]

Table S2. The number of contacts while having food (mean, median, SD) younger, same age or older than participant.

Age group	Younger	Same age	Older
14-19	12.4 (0; 27.8)	27.3 (15; 33.5)	27.3 (12; 29.9)
20-29	1.5 (0; 3.4)	6.1 (3; 9.5)	4.6 (2; 7.8)
30-39	4.4 (3; 4.1)	3.0 (1; 5.1)	3.0 (2; 3.9)
40+	5.0 (4; 5.4)	3.0 (0; 5.8)	3.8 (2; 5.7)

Note: Number of contacts while eating was censored to a maximum of 75 contacts per day for each respondent.
