# Supplementary material for: Online Respondent-Driven Sampling for Studying Contact Patterns Relevant for the Spread of Close-Contact Pathogens: A Pilot Study in Thailand
Source: PLoS One. 2014 Jan 8;9(1):e85256. doi: 10.1371/journal.pone.0085256 (PMC3885693; doi:10.1371/journal.pone.0085256)
Supplement: Table S3 — Reported symptoms. (PDF) [file pone.0085256.s005.pdf]

Table S3. Reported symptoms.

	Freq.	mean (SD) number of household contacts with symptoms[a]	Did not know whether household contacts had one or more related symptoms[b]
Fever	25	0.48 (0.87)	0
Chills	10	1.50 (1.51)	0
Runny nose	72	0.76 (1.28)	6
Sore throat	59	0.79 (1.13)	7
Cough	39	0.53 (0.88)	4
Headache	83	2.04 (3.25)	10
Muscle pain	88	1.02 (1.39)	7
Diarrhea	18	0.69 (1.14)	2
Other symptoms	6	0.50 (0.84)	0
No symptoms	59	0.79 (1.49)	6

a) Household contacts having one or more related symptoms. **b)** Number of participants who reported the symptom, but did not know whether household members also had one or more related symptoms.
