# Supplementary material for: Online Respondent-Driven Sampling for Studying Contact Patterns Relevant for the Spread of Close-Contact Pathogens: A Pilot Study in Thailand
Source: PLoS One. 2014 Jan 8;9(1):e85256. doi: 10.1371/journal.pone.0085256 (PMC3885693; doi:10.1371/journal.pone.0085256)
Supplement: Text S1 — Fitting of a negative binomial distribution to degree. (PDF) [file pone.0085256.s006.pdf]

Text S1. Fitting of a negative binomial distribution to degree.

Degree was defined as the sum of the numbers of contacts while travelling and at different locations reported by each respondent for one day (Figure Ia). We observed no assortativeness (i.e. random mixing) by degree between linked nodes (Figure Ib). We investigated whether the observed distribution agreed with a random mixing in a population with a negative binomially distributed degree, by fitting a theoretical distribution to the empirical degree distribution.

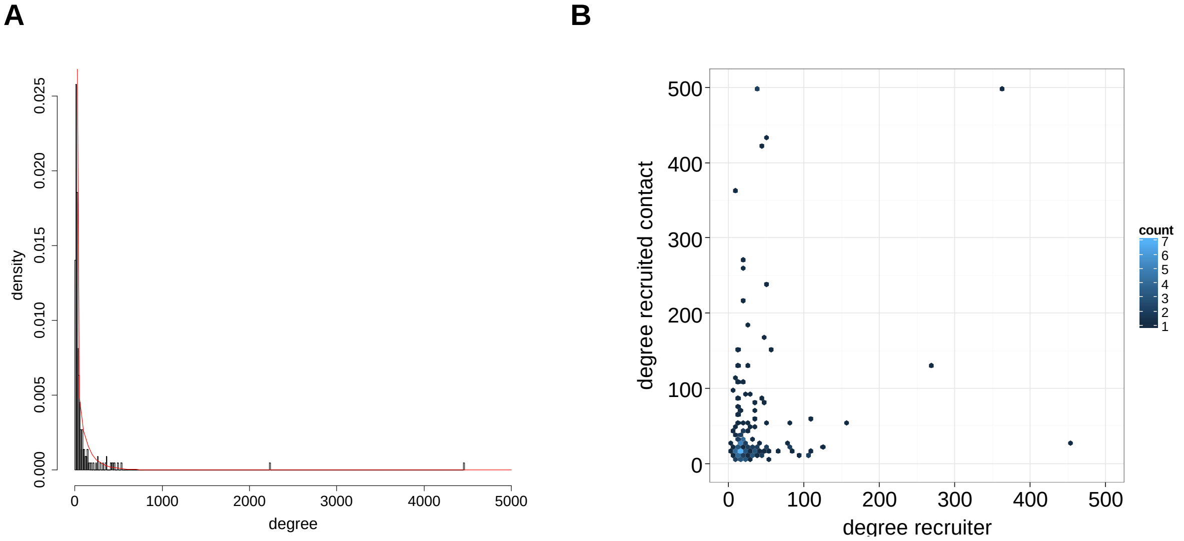

Figure I. Distribution of degree. A. Distribution of reported degree, the red line indicates the fitted negative binomial distribution. **B.** Correlations between degree recruiter and recruited contact. Overlapping points were made visible with a colour scale.

The count variable degree exhibited extreme overdispersion, i.e. the sample variance (117028.2) highly exceeded the mean (88.24), making the negative binomial (NB) distribution an appropriate model for fitting [1]. We fitted an NB distribution to the observed degree distribution using maximum likelihood (ML) estimation in R (version 2.5.3). In R, the mean number of counts (μ) in a sample is defined as *mu*, and the overdispersion parameter (k) is defined as *size*. The overdispersion parameter measures the amount of heterogeneity (or clustering) in the data. A smaller k means more heterogeneity (i.e. as k becomes large the variance approaches the mean and the distribution approaches the Poisson distribution) [2].

ML estimates for degree: *mu* = 88.2; k = 0.57. We did not assess statistically the fit of the NB distribution to the observed degree distribution. As was shown earlier by Lloyd-Smith (2007), small-sample estimates of k can be biased towards overestimating k and consequently to underestimation of the level of overdispersion in the data, when using maximum likelihood. Smaller samples are less likely to include values from the right-hand tail of the NB distribution, and without these outliers a dataset appears to be more homogeneous [3].

Literature

1. Bliss CI, Fisher RA (1953) Fitting the negative binomial distribution to biological data - note on the efficient fitting of the negative binomial. Biometrics 9: 176–200.

2. Bolker B (2007) Probability Distributions: Negative binomial. Ecological Models and Data in R. Princeton and Oxford: Princeton University Press.
3. Lloyd-Smith JO (2007) Maximum likelihood estimation of the negative binomial dispersion parameter for highly overdispersed data, with applications to infectious diseases. PLoS One 2: e180.
