# Supplementary material for: Online Respondent-Driven Sampling for Studying Contact Patterns Relevant for the Spread of Close-Contact Pathogens: A Pilot Study in Thailand
Source: PLoS One. 2014 Jan 8;9(1):e85256. doi: 10.1371/journal.pone.0085256 (PMC3885693; doi:10.1371/journal.pone.0085256)
Supplement: Text S2 — Links between recruiter and recruited contact person: descriptive statistics. (PDF) [file pone.0085256.s007.pdf]

Text S2. Links between recruiter and recruited contact person: descriptive statistics.

We studied the correlations between recruiter and his/her recruited contact for different characteristics. This supplementary text file provides an overview of the descriptive statistics from which the correlation coefficients shown in Tables 3 and 4 (see main article) were estimated. The scatterplots for the variables age and degree are displayed in the manuscript. Count data was log transformed for conducting null hypothesis tests.

<u>Gender:</u> Table I shows the absolute number of females/males who recruited other females/males. In total, 166 respondent-contact person pairs provided their gender. 57.8% was female and 42.2% male.

Table I. Who recruited whom? Gender

	**Gender recruited contact**	
**Gender recruiter**	female	male
female	71 (42.8%)	38 (22.9%)
male	25 (15.1%)	32 (19.3%)

<u>Degree:</u> Degree was calculated as the sum of the number contacts while travelling and at different locations (Figure I). Both the log distribution (Figure Ib) and the joint probability distribution of log degree visually approximated a normal distribution. There were three respondents who reported more than 500 contacts for the particular recording day (namely 533, 2233, 4456 contacts) that were censored to a maximum of 500 contacts.

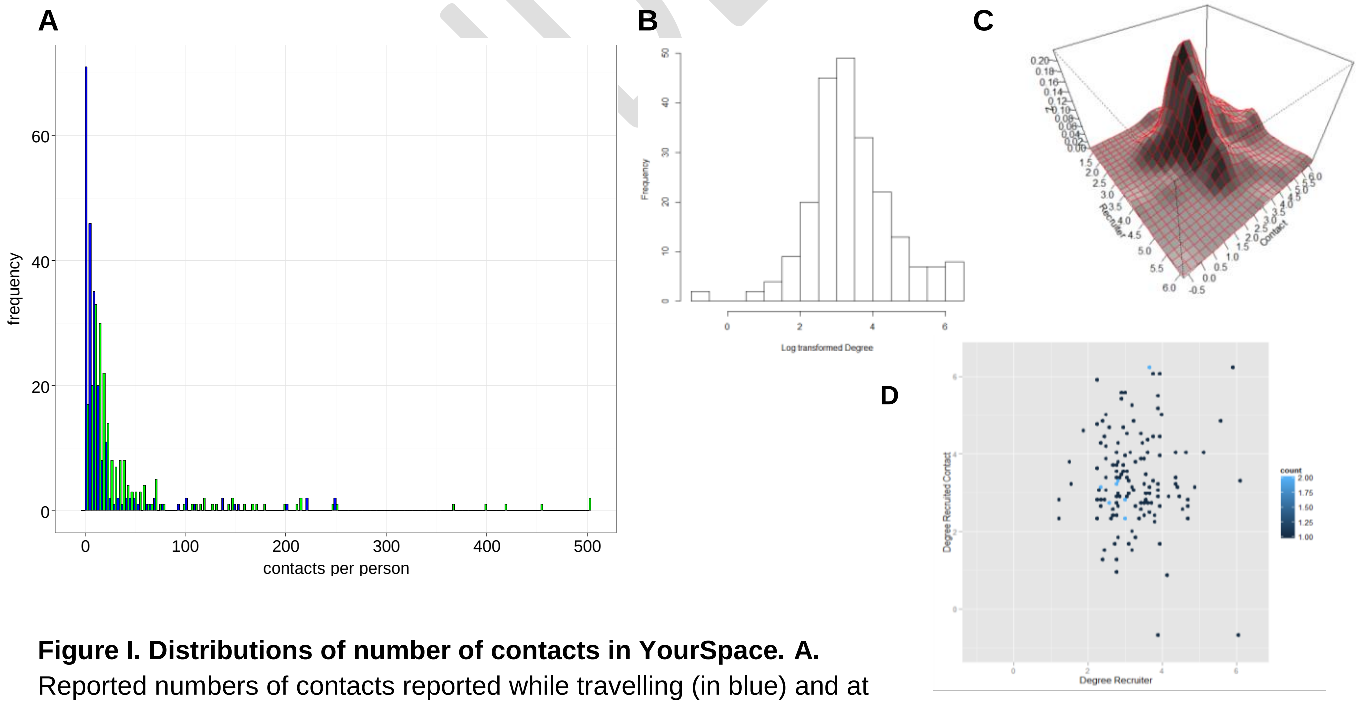

Figure I. Distributions of number of contacts in YourSpace. A. Reported numbers of contacts reported while travelling (in blue) and at different locations (in green), untransformed. **B.** Distribution of log degree. Degree is the sum of the two distributions displayed in graph A. **C.** The joint probability distribution of log degree (recruiter versus contact) approximated bivariate normality. **D.** Scatterplot of log degree recruiter versus log degree of his/her recruited contact, which shows random mixing by degree.

Household size: Household size was defined the number of persons living the household of the respondent in the seven days prior to the recording day. The log distribution of household size did not approximate a normal distribution (as shown in Figures IIa and IIb) due to a large number of respondents (>40) who reported only a one-person household. Figures IIc and IId showed random mixing by household size.

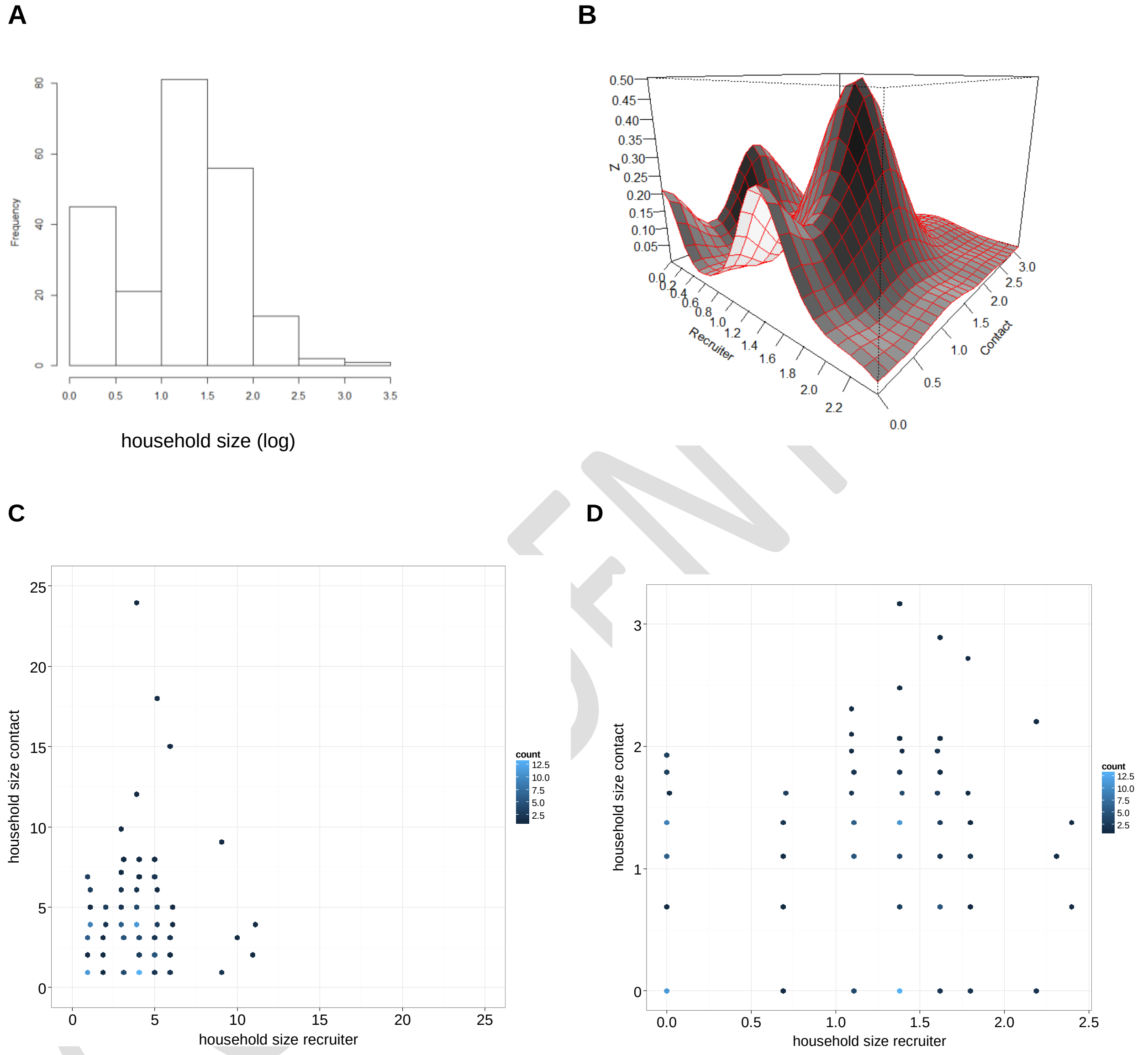

Figure II. Distribution of household size. A. Distribution of household size, log transformed. **B.** The joint probability distribution of log household size (recruiter versus contact). The distribution did not approximate bivariate normality due to >40 respondents who reported only one-person households. **C.** Scatterplot of household size recruiter versus household size of his/her recruited contact, untransformed. **D.** Scatterplot of log household size versus log household size of his/her recruited contact.

<u>Contacts while eating:</u> We asked participant to report the number of contacts (according to the contact definition) while having breakfast, lunch, dinner and snack break. There were four respondents who reported in total more than 75 contacts for the particular recording day (namely 76, 125, 376 and 1086 contacts). These were censored to a total maximum of 75 contacts while eating. The log distribution of contacts while eating approximated a normal distribution (see Figures IIIa and IIIb).

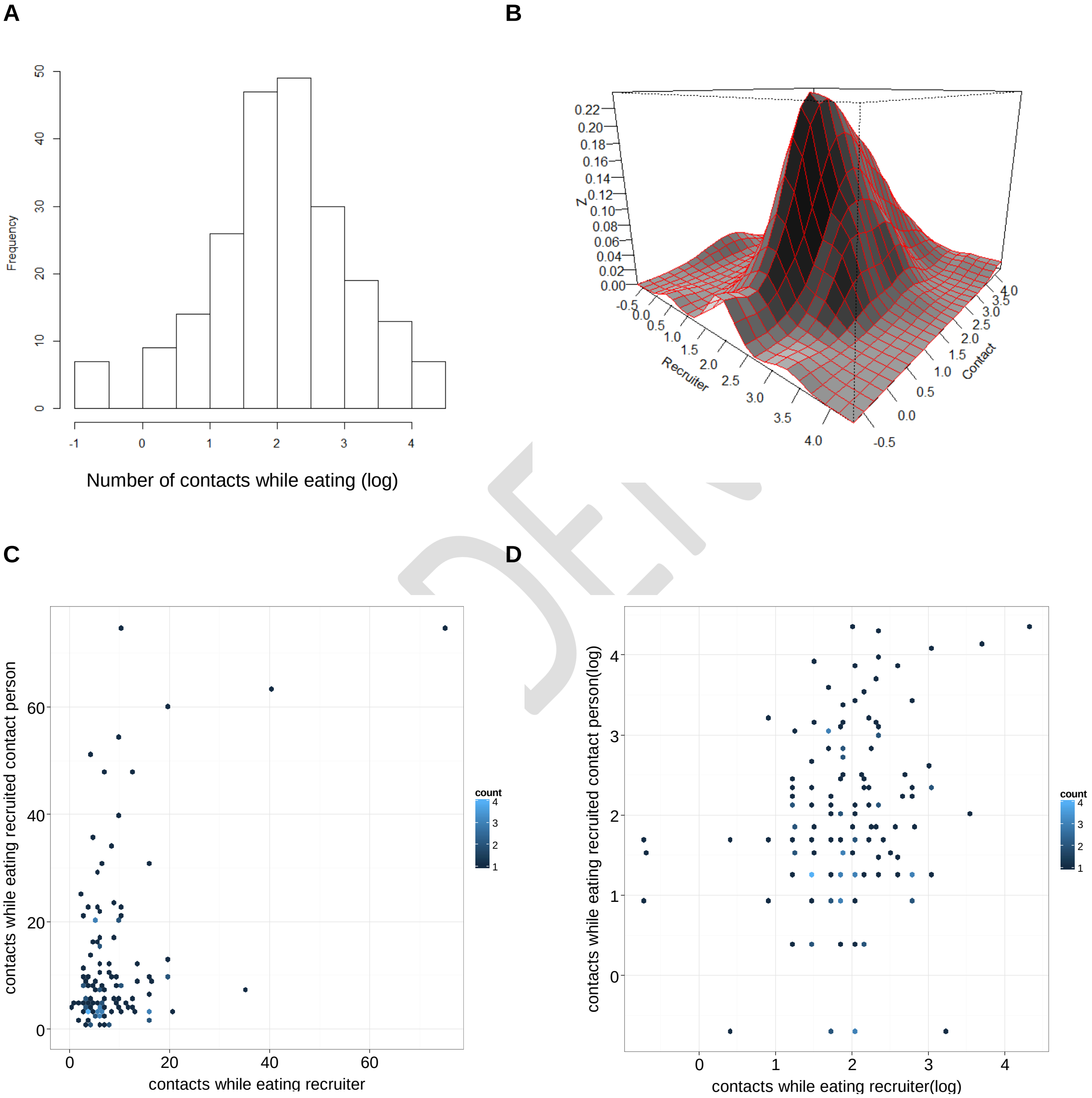

Figure III. Distribution of contacts while eating. A. Distribution of numbers of contacts while eating, log transformed. **B**. The joint probability distribution of log contacts while eating (recruiter versus contact). This distribution approximated bivariate normality. **C.** Scatterplot of numbers of contacts while eating of recruiter versus his/her recruited contact person, untransformed. **D.** Scatterplot of log numbers of contacts while eating of recruiter versus his/her recruited contact person.

<u>Number of self-reported symptoms:</u> Self-reported influenza-like-illness (ILI) symptoms were collected by providing respondents with a list of nine symptoms. For each respondent the number of symptoms reported was summed. The log distribution did not approximate a normal distribution due to the large proportion of respondents who reported no symptoms (Figures IVa and IVb). Table S3 contains the reported numbers for each symptom separately.

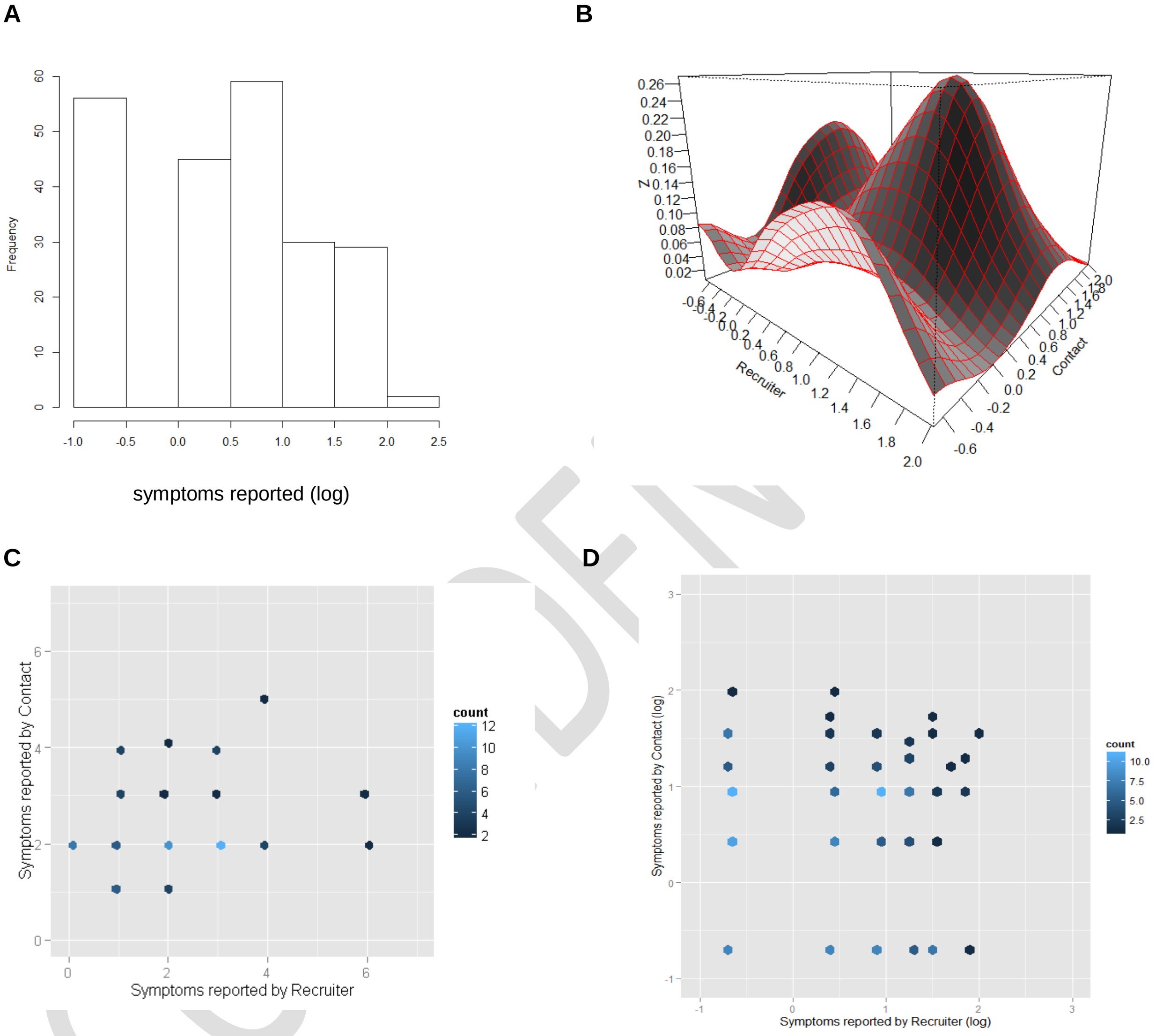

Figure IV. Distribution of self-reported symptoms. A. Distribution of numbers of reported symptoms, log transformed. **B**. The joint probability distribution of log reported symptoms. This distribution did not approximate bivariate normality. **C.** Scatterplot of numbers of reported symptoms by recruiter versus his/her recruited contact person, untransformed. **D.** Scatterplot of log numbers of reported symptoms by recruiter versus his/her recruited contact person.
