# Supplementary material for: Online Respondent-Driven Sampling for Studying Contact Patterns Relevant for the Spread of Close-Contact Pathogens: A Pilot Study in Thailand
Source: PLoS One. 2014 Jan 8;9(1):e85256. doi: 10.1371/journal.pone.0085256 (PMC3885693; doi:10.1371/journal.pone.0085256)
Supplement: Text S3 — Drivers of the recruitment process. (PDF) [file pone.0085256.s008.pdf]

Text S3. Drivers of the recruitment process.

To investigate which variables are important in the recruitment process, we analysed **A.** the intention to recruit (categorised as “respondent requested” OR “did not request” four invitations for contacts on the last survey page) and **B.** the number of contact persons successfully recruited (categorised into “zero” OR “one or more contact persons”) by each respondent, using logistic regression.

Confidence intervals for the coefficient estimates were obtained using standard errors. We also used a Pearson’s Chi-Squared test (with Yates’ continuity correction) to analyse bivariate the independency between the outcome and the categorical predictors. In addition, we investigated how well the model with all predictors fits compared to a null model (i.e. a model with just an intercept). The test statistic is the difference between the residual deviance for the model with predictor and the null model, and is distributed chi-squared with degrees of freedom (df) equal to the differences in df between the model with all predictors and the null model.

Note that our sample size is relatively small for the number of predictor variables that we are analysing, which could result in an unstable model. We produced contingency tables to check for empty cells or cells with a low number of observations (Tables I and IV). In addition, observations are not independently collected with a respondent-driven sampling method, thereby violating a condition for conducting logistic regression.

A. Intention to recruit contact persons

We performed a logistic regression with the intention to recruit as binary outcome, categorised as “a respondent did or did not request for four invitations on the last survey page” (n = 220). The log odds of the outcome was modeled as a linear combination of the variables: age (integer), gender (binary), education (ordinal), household size (integer) and degree (integer, i.e. number of contacts).

Table I. Contingency table of categorical outcome and predictor variables.

		**Education**		
**Intention to recruit**	**Gender**	Lower than bachelor	Bachelor	Master
No	Female	9	48	9
	Male	8	27	8
Yes	Female	10	55	24
	Male	14	29	12
	**Total**			*253*

Table II. Chi-squared test to analyse independence by outcome variable.

	**Chi-squared**	**df**	**p-value**
Gender	0.0053	1	0.9421
Education	3.7457	2	0.1537

Table II shows that the outcome “intention to recruit” is independent of gender and education. The logistic regression coefficients in Table III give the change in log odds of the outcome for one unit increase in the predictor variable. None of the included variables have a statistically significant effect on the outcome, which indicates that none of these variables significantly increase (or decrease) the intention to recruit contact persons by a respondent. The Chi-

Square of 8.64 with 6 df and an associated p-value of more than 0.05 (0.1950) showed that the model with predictors does not fit significantly better than the null model, which is in agreement with the output from Table III.

Table III. Output of logistic regression with as binary outcome 'intention to recruit' (A).

	Estimate[a]	SE	z value	Pr(>|z|)	2.5%	97.5%
Constant	0.2414	0.7318	0.3298	0.7415	-1.1929	1.6757
Age	0.0263	0.0261	1.0103	0.3123	-0.0248	0.0774
Male	0.1166	0.3075	0.3791	0.7046	-0.4862	0.7193
Edu Bachelor	-0.6747	0.4534	-1.4881	0.1367	-1.5634	0.2140
Edu Master	-0.0937	0.5792	-0.1618	0.8714	-1.2289	1.0414
Household size	0.0632	0.0506	1.2491	0.2116	-0.0360	0.1623
Degree (number of contacts)	-0.0022	0.0015	-1.4696	0.1417	-0.0051	0.0007

[a]Null deviance: 283.62 (df: 219); residual variance: 274.98 (df: 213) and AIC: 288.98.

B. Number of contact persons successfully recruited

The number of contact persons successfully recruited by each respondent, i.e. contact person also completed the questionnaire, was divided into "zero" or "one or more contact persons" (*n* = 144). The log odds of this binary outcome (yes/no recruited contact persons) was modelled as a linear combination of the variables: age, gender, education, household size, degree and recruitment option used (binary: Facebook or email).

Table IV. Contingency table of categorical outcome and predictor variables.

			**Recruitment option used**	
**Recruited**	**Gender**	**Education**	Facebook	Email
No	Female	Lower than bachelor	2	2
		Bachelor	20	6
		Master	7	4
	Male	Lower than bachelor	6	0
		Bachelor	14	2
		Master	4	0
Yes	Female	Lower than bachelor	6	0
		Bachelor	25	4
		Master	9	4
	Male	Lower than bachelor	7	1
		Bachelor	9	4
		Master	7	1
		**Total**		144

Table V. Chi-squared test to analyse independence by outcome variable

	Chi-squared	df	p-value
Gender	0.0084	1	0.9271
Education	3.5934	2	0.1658
Recruitment option used	0.0397	1	0.842

Table V shows that the outcome variable "successfully recruited" is independent of gender, education and recruitment option. The logistic regression coefficients in Table VI give the change in log odds of the outcome for one unit increase in the predictor variable. Of all variables, only household size seems to significantly influence recruitment. For one unit increase in household size, the log odds of successfully recruiting contact persons decreases by -0.1527. However, running a logistic model that only contains household size as a predictor variable does not show a significant effect by this variable. In addition, the Chi-Square of 8.41 with 7 df and an associated p-value of more than 0.05 (0.2980), showed that the model with predictors does not fit significantly better than the null model. The significant influence by household size seen in Table VI is probably caused by the low number of observations (empty cells in the contingency table, see Table IV) that make the model unstable, or due to interference between included predictor variables.

Table VI Output of logistic regression with as binary outcome successfully recruited yes/no.

	Estimate[a]	SE	z value	Pr(>|z|)	2.5%	97.5%
Constant	1.6780	0.8664	1.9368	0.0528	-0.0201	3.3760
Age	-0.0316	0.0301	-1.0467	0.2952	-0.0906	0.0275
Male	-0.0925	0.3631	-0.2546	0.7990	-0.8041	0.6192
Edu Bachelor	-0.1954	0.5062	-0.3860	0.6995	-1.1876	0.7968
Edu Master	0.1946	0.6340	0.3070	0.7588	-1.0480	1.4373
Household size	-0.1527	0.0699	-2.1859	0.0288	-0.2897	-0.0158
Degree (number of contacts)	0.0012	0.0020	0.6109	0.5412	-0.0027	0.0052
Email	-0.0564	0.4536	-0.1243	0.9011	-0.9455	0.8327

[a]Null deviance: 198.93 (df: 143); residual variance: 190.52 (df: 136) and AIC: 206.52.
