# Supplementary material for: Online Respondent-Driven Sampling for Studying Contact Patterns Relevant for the Spread of Close-Contact Pathogens: A Pilot Study in Thailand
Source: PLoS One. 2014 Jan 8;9(1):e85256. doi: 10.1371/journal.pone.0085256 (PMC3885693; doi:10.1371/journal.pone.0085256)
Supplement: Text S4 — Exploring the first–order Markov assumption. (PDF) [file pone.0085256.s009.pdf]

Text S4. Exploring the first–order Markov assumption.

A respondent-driven (i.e. chain referral) sample can be viewed as a stochastic process in which the social characteristics of each recruiter affect the characteristics of the recruited contact persons. If the sampling conforms to methodological requirements, the proportion of the sample with a certain characteristic is expected to stabilise at a level determined by the characteristics of the population and independent of the characteristics of the seeds [1]. The recruitment process can be modelled as a Markov process, in which the process can assume a limited number of states and is state dependent (i.e. the probability that the recruited contact person comes from a given group depends on the group from which the current recruiter comes). If the recruitment patterns depend only on the recruiter and not on the recruiter's recruiter (or recruiters before that) the recruitment chain corresponds to a first–order Markov process [2]. Although our sample contained only a limited number of waves, we explored whether the resulting recruitment chains are consistent with a first–order Markov process by comparing correlations for age, gender and education found in our sample data with correlations obtained from simulated data.

In the main text we showed the correlations between any two respondents with different link distances in the same network tree (see Table 3 and Figure 4), using Dijkstra's algorithm (which calculates shortest paths between any two persons in the same network tree). The geodesic distance between two nodes (or vertices) is the number of edges in a shortest path connecting them.

To analyse whether recruitment by recruiters in wave 1 and higher is dependent on the seed, we first calculated the correlations between seeds (wave 0) and their contact persons in consecutive waves (maximum up to 3 waves, due to limited number of respondents in the waves ≥4). We then used Monte Carlo techniques to simulate (with n=10.000) a first–order autoregressive process (i.e. an autoregressive Markov Chain of order one) based on the correlation found in the sample data between seeds and their contact persons in wave 1. For this, we used the function 'arima.sim' of the R–package 'stats' (version 2.15.3). We compared correlations estimated from the sample data (r) with correlations estimated from the simulated data (rr) with the same geodesic distance.

Table I shows that the sample correlations (r) for age and gender, decreased slower than the simulated correlations (rr) over the same geodesic distances. For example, for age, the sample correlation between seeds in wave 0 and contact persons in wave 2 is r = 0.554, while rr = 0.277. This suggests that the recruitment process is a higher order process with regard to the variables age and gender. Thus, correlations found between the recruited contact persons and their recruiter are not only dependent on the direct recruiter, but also on recruiters in previous waves. Due to the limited number of waves in most of our network trees, we were unable to quantify the exact order level. For education, correlations for the geodesic distances 1 and 2 were lower in the data than in the simulations, suggesting a first-order Markov process (i.e. education of contact persons are only dependent on the education of their direct recruiter).

Table I. Correlations between seeds and contact persons in waves 1 to 3, estimated from sample data and simulated data.

		Wave 0	
	**Geodesic distance**	**Correlations sample data** (*r*)	**Correlations simulated data** (*rr*)
**Age** (*r*)	**Wave 1**	0.524 [0.350–0.663][a]	0.527 [0.512–0.541]
	**Wave 2**	0.554 [0.304–0.732][b]	0.277 [0.259–0.295]
	**Wave 3**	0.320 [-0.143–0.668][c]	0.150 [0.131–0.169]
**Gender** (r_{φ})	**Wave 1**	0.192 [-0.022–0.389][a]	0.204 [0.185–0.223]
	**Wave 2**	0.172 [-0.135–0.450][b]	0.057 [0.037–0.077]
	**Wave 3**	0.000 [-0.443–0.443][c]	0.024 [0.004–0.044]
**Education** (r_{rank})	**Wave 1**	0.701 [0.559–0.811][a]	0.677 [0.664–0.688]
	**Wave 2**	0.224 [-0.09–0.519][b]	0.465 [0.448–0.481]
	**Wave 3**	0.000 [-0.167–0.192][c]	0.331 [0.314–0.348]

a) n_{pairs} = 85; **b)** n_{pairs} = 43; **c)** n_{pairs} = 20

References

1. Heckathorn DD (2002) Respondent–Driven Sampling II: Deriving Valid Population Estimates from Chain–Referral Samples of Hidden Populations. Social Problems 49: 11–34.
2. Heckathorn D (1997) Respondent–driven sampling: a new approach to the study of hidden populations. Social Problems 44: 174–199.
