# Supplementary material for: Online Respondent-Driven Sampling for Studying Contact Patterns Relevant for the Spread of Close-Contact Pathogens: A Pilot Study in Thailand
Source: PLoS One. 2014 Jan 8;9(1):e85256. doi: 10.1371/journal.pone.0085256 (PMC3885693; doi:10.1371/journal.pone.0085256)
Supplement: Text S5 — Sample composition, equilibrium curves and RDS estimates. (PDF) [file pone.0085256.s010.pdf]

Text S5. Sample composition, equilibrium curves and RDS estimates.

To assess whether the sample reached equilibrium (i.e. as the sample grows in size, the composition of the sample ceases to change [1]), we plotted the sample composition over waves for gender, education and age (see Figure S1) and visually assessed whether the process reached equilibrium. The curves in Figure S1 indicate that equilibrium was reached for gender, education and age after a maximum of three waves. However, the achievement of equilibrium over waves for these variables is greatly influenced by the relatively small sample size and the limited number of waves in most network trees (most trees had a maximum of one or two waves).

We also plotted the sample proportion by increasing sample sizes for all variables surveyed in this study (see Figure S2). We used the Volz-Heckathorn (VH) estimator (or 'RDSII' estimator) to provide RDS corrected estimates, thus, the proportion of individuals with characteristic *A* in the population. The VH-estimator requires only information on the sample compositions and the personal network sizes of the participants. Degree (number of reported contacts) was used to define the participants' personal network size. The Volz-Heckathorn estimator is defined as [2]:

$$\hat{P}_A^{VH} = \frac{\sum_{i \in A \cap S} d_i^{-1}}{\sum_{i \in S} d_i^{-1}}$$

Where d_i is the degree of individual *i*, and *S* the set of sampled individuals.

For the variables age (Figure S2E), gender (Figure S2F), education (Figure S2A) and recruitment option (Figure S2C), where the number of observations (*n*) were 253, we replaced missing degree data with the VH-estimate for degree (average). The VH-estimate for degree was calculated by all those participants who reported a degree (participants with non-missing network data). Degree was censored to a maximum of 500 contacts per day per individual. The VH estimate for degree was 17.3 contacts per participant per day. For all other variables, we removed missing data and used only data from completely filled in questionnaires (*n*=220).

Judging from the plots in Figure S2, the sample composition did not stabilise for most variables, with the exception of household size, household members with symptoms, contacts while eating and self-reported symptoms, which seem to have stabilised after 200 participants.

All curves in Figure S2 show sudden increases or decreases in VH-estimates along with increasing sample sizes. For variables that are calculated in proportions, these jumps are related to individuals who reported either a high degree (e.g. more than 100 contacts, resulting in a decrease in VH-estimate) or a low degree (e.g. less than 5 contacts, resulting in an increase in VH-estimate). Similar for age and other variables calculated in averages, participants with a high age (e.g. >40, see Figure S2E) or a high number of contacts (e.g. contacts while eating, see Figure S2G) result in sudden increases or decreases in VH-estimates.

Table I contains the sample means and VH estimates for all variables, estimated over the whole sample. Our sample mainly represents students and their contacts of similar age, and only those individuals that have access to the internet and those who use Facebook (the age

groups 20-39 use the internet the most in Thailand [3]). Especially the age group 20-35 years, females and those with a bachelor or master degree are strongly overrepresented in our sample, compared to available Thai demographical estimates, which influenced the VH-estimates. For example, the VH-estimator estimated based on our sample that 82.9% of the Thai population was between 20-34 years, while the actual proportion is estimated to be 22.6% [4]. However, according to the VH-estimate the average household size in Thailand is 3.52, which is close to the average of 3.2 provided by the National Statistical Office Thailand [4]. Also, the VH-estimates for the different household sizes in Thailand were similar to the proportions provided by the ETDA Thailand [3], especially for the household sizes 4, 5 and 6 or more (Table I).

The overrepresentation of certain individuals is likely because we invited only students to act as seeds in our RDS survey, and the number of obtained waves was insufficient for our survey to penetrate into different layers of the Thai population. Although the internet-use (and access) is not equally divided over all age groups in Thailand, a future webRDS survey with a higher number of waves and with seeds selected from different age groups could possibly provide a better representative sample for the entire Thai population.

Table I. Sample proportions and estimated population proportions over the whole sample.

	Category	*n*	Sample mean	VH-estimate	Demographics Thailand[a]
Age (average)		253	26.70 (median: 25.00)	27.85	Median: 35.1 (2013) [5]
Age groups (%)	10-14	253	0.79	0.19	6.45%[b] [6]<br>IU[c]: (<15): 0.8% [3]
	15-19		3.16	1.44	7.51%[b] [6]<br>IU[c]: 5.8% [3]
	20-24		39.13	40.57	7.25%[b] [6]<br>IU[c]: 14.9% [3]
	25-29		32.02	29.33	7.32%[b] [6]<br>IU[c]: 15.9% [3]
	30-34		13.83	13.02	8.02%[b] [6]<br>IU[c]: 18.0% [3]
	35-39		5.93	7.28	8.19%[b] [6]<br>IU[c]: 14.7% [3]
	40-44		1.19	1.95	8.37%[b] [6]<br>IU[c] (40-49):19.4% [3]
	45-49		2.37	1.97	7.93%[b] [6]
	50-54		1.58	4.24	6.85[b] [6]<br>IU[c] (50-59): 9.8% [3]
Male (%)		253	38.74	35.00	49.18 (2010) [7]
Education (%)	J. High S.	253	0.40	0.17	4.07%[b] [8]
	High School		15.02	9.39	3.25%[b] [8]
	Higher Dipl.		0.79	0.45	3.71%[b] [8]
	Bachelor		62.85	67.34	
	Master		20.95	22.65	
Household size (average)		220	4.11	3.52	3.2 (2010) [4]
Household size, categorized (%)	1	220	20.45	22.57	14.7% [3]
	2		9.55	12.28	15.9% [3]
	3		16.36	15.47	19.2% [3]
	4		20.45	25.28	24.2% [3]
	5		14.09	13.03	13.7% [3]
	6 or more		19.09	11.37	12.3% [3]
Proportion used recruitment option (%)	Facebook	253	45.85	40.00	18.5 million users [9]: 28.70%[b]
	Indirect email		5.53	9.76	
	Direct email		5.53	3.92	
	No recruitment		43.08	46.32	

Average number of contacts while eating (%)		220	12.21	6.90	
Proportion household members with symptoms (%)	0	220	57.27	52.78	
	1		15.45	11.02	
	2		8.64	8.13	
	3		3.64	5.25	
	4		3.18	2.28	
	5		2.27	5.29	
	6 or more		0.45	0.49	
	Unknown		9.09	14.75	
Number of symptoms (average)		220	1.80	1.63	
Flu/Cold Symptoms (%)	Flu symptoms	220	2.73	1.69	
	Cold symptoms		5.91	5.33	

a) Population size Thailand: 64456695 (2012) [6]. **b)** Calculated with actual number of individuals in each group and total population Thailand. **c)** Internet-using (IU) population.

Literature

1. Heckathorn DD, Semaan S, Broadhead RS, Hughes JJ (2002) Extensions of Respondent-Driven Sampling: A New Approach to the Study of Injection Drug Users Aged 18–25. AIDS and Behavior 6: 55-67.
2. Volz E, Heckathorn DD (2008) Probability based estimation theory for respondent driven sampling. Journal of Official Statistics 24: 79-97.
3. Electronic Transactions Development Agency (Public Organization), Ministry of Information and Communication Technology (2013) Thailand Internet User Profile 2013.
4. National Statistical Office, Ministry of Information and Communication Technology Thailand (2012) The Information and Communication Technology Survey in Household.
5. Central Intelligence Agency (2013) The World Factbook. 25 October 2013 ed.
6. Statistical Forecasting Bureau, National Statistical Office Thailand, Ministry of Information and Communication Technology (2013) Statistical Yearbook Thailand 2013.
7. National Statistical Office, Ministry of Information and Communication Technology Thailand (2012) Key statistics of Thailand 2012.
8. Statistical Forecasting Bureau, National Statistical Office (2013) Thailand's Key Indicators 2013.
9. Socialbakers (2013) Checkfacebook: Facebook statistics of Thailand.
